# Systemic and cerebrospinal fluid immune and complement activation in Ugandan children and adolescents with long‐standing nodding syndrome: A case‐control study

**DOI:** 10.1002/epi4.12463

**Published:** 2021-03-12

**Authors:** Rodney Ogwang, Dennis Muhanguzi, Kioko Mwikali, Ronald Anguzu, Joe Kubofcik, Thomas B. Nutman, Mark Taylor, Charles R. Newton, Angela Vincent, Andrea L. Conroy, Kevin Marsh, Richard Idro

**Affiliations:** ^1^ Makerere University College of Health Sciences Kampala Uganda; ^2^ Centre of Tropical Neuroscience (CTN) Kitgum Site Uganda; ^3^ KEMRI‐Wellcome Trust Research Programme Centre for Geographic Medicine Coast Kilifi Kenya; ^4^ College of Veterinary Medicine Animal Resources and Biosecurity Makerere University Kampala Uganda; ^5^ Division of Epidemiology Institute of Health and Equity Medical College of Wisconsin Wisconsin WI USA; ^6^ Laboratory of Parasitic Diseases National Institute of Allergy and Infectious Diseases Bethesda MD USA; ^7^ Liverpool School of Tropical Medicine Liverpool UK; ^8^ Department of Psychiatry University of Oxford Oxford UK; ^9^ Nuffield Department of Clinical Neurosciences University of Oxford Oxford UK; ^10^ Indiana University School of Medicine Ryan White Center for Pediatric Infectious Disease & Global Health Indianapolis IN USA; ^11^ Centre for Tropical Medicine and Global Health Nuffield Department of Medicine University of Oxford Oxford UK

**Keywords:** chemokine, cytokine, Epilepsy, neuroinflammation, Northern Uganda

## Abstract

**Objective:**

Nodding syndrome is a poorly understood epileptic encephalopathy characterized by a unique seizure type—head nodding—and associated with *Onchocerca volvulus* infection. We hypothesized that altered immune activation in the cerebrospinal fluid (CSF) and plasma of children with nodding syndrome may yield insights into the pathophysiology and progression of this seizure disorder.

**Method:**

We conducted a case‐control study of 154 children (8 years or older) with long‐standing nodding syndrome and 154 healthy age‐matched community controls in 3 districts of northern Uganda affected by nodding syndrome. Control CSF samples were obtained from Ugandan children in remission from hematological malignancy during routine follow‐up. Markers of immune activation and inflammation (cytokines and chemokines) and complement activation (C5a) were measured in plasma and CSF using ELISA or Multiplex Luminex assays. *O* *volvulus* infection was assessed by serology for anti–OV‐16 IgG levels.

**Results:**

The mean (SD) age of the population was 15.1 (SD: 1.9) years, and the mean duration of nodding syndrome from diagnosis to enrollment was 8.3 (SD: 2.7) years. The majority with nodding syndrome had been exposed to *O* *volvulus* (147/154 (95.4%)) compared with community children (86/154 (55.8%)), with an OR of 17.04 (95% CI: 7.33, 45.58), *P* < .001. C5a was elevated in CSF of children with nodding syndrome compared to controls (*P* < .0001). The levels of other CSF markers tested were comparable between cases and controls after adjusting for multiple comparisons. Children with nodding syndrome had lower plasma levels of IL‐10, APRIL, CCL5 (RANTES), CCL2, CXCL13, and MMP‐9 compared with community controls (*P* < .05 for all; multiple comparisons). Plasma CRP was elevated in children with nodding syndrome compared to community children and correlated with disease severity.

**Significance:**

Nodding syndrome is associated with exposure to *O. volvulus*. Compared to controls, children with long‐standing symptoms of nodding syndrome show evidence of complement activation in CSF and altered immune activation in plasma.

**Key points**

- Long‐standing nodding syndrome may be associated with complement activation in CSF.
- The pathogenesis of chronic nodding syndrome may involve systemic and CSF immune alterations.
- *Onchocerca volvulus* infection is associated with nodding syndrome, but its role in disease pathogenesis remains inconclusive.

## 1 INTRODUCTION

Nodding syndrome is a poorly understood severe neurological disorder characterized by a unique seizure manifesting as repeated head nods and complicated by other generalized seizures, motor, neuropsychiatric, and cognitive impairments.^1^, ^2^ In addition, undernutrition, delayed development of sexual characteristics, and stunting are observed in a proportion of affected children. Nodding syndrome was initially reported in southern Tanzania, followed by South Sudan and later in northern Uganda.^3^, ^4^, ^5^ More recently, cases have been reported in Central Africa.^6^ Nodding syndrome occurs in previously normal children between the ages of 3 and 18 years, and it is chronic, shows progression in some, and frequently occurs in epidemics with clusters of cases observed in geographically distinct regions.^7^, ^8^ Clinical and electrophysiological studies suggest nodding syndrome may be an epileptic encephalopathy.^9^

The etiology of nodding syndrome remains unknown. It has been proposed that nodding syndrome may be linked to a nutritional, genetic, or an unknown infectious agent.^10^, ^11^ However, several epidemiological studies have demonstrated a consistent relationship with *O volvulus* infection leading to the hypothesis that *O volvulus* may underlie the pathogenesis of nodding syndrome.^5^, ^12^, ^13^, ^14^ Confounding this hypothesis is the fact that *O volvulus* is observed across Africa and in parts of Latin America,^15^, ^16^ whereas nodding syndrome is largely confined to East and Central Africa.^17^ In addition, a proportion of healthy community controls typically have evidence of *O volvulus* infection. Despite this, recent evidence suggests a link between seizure‐related disorders and *O volvulus* leading to the study of a spectrum of syndromes—Onchocerca‐associated epilepsies (OAE). The mechanism by which *O volvulus* infection may lead to nodding syndrome and/or the spectrum of disorder labeled as OAEs remains unclear. One school of thought has suggested that *O volvulus* may be a secondary opportunistic infection unrelated to the etiology of nodding syndrome.^18^ It has also been hypothesized that antibodies to *O volvulus* or its symbiont *Wolbachia* may cross‐react with neuronal proteins leading to neuroinflammation and nodding syndrome.^13^ This hypothesis is supported by studies showing auto‐antibodies against actin‐binding protein leiomodin‐1,^19^ and the voltage‐gated potassium channel complex^13^ in the cerebrospinal fluid (CSF) of patients with nodding syndrome. A recent postmortem study of brains from patients with nodding syndrome demonstrated evidence of past ventriculitis, meningitis, and gliotic lesions suggesting nodding syndrome may be a postinfectious encephalopathy.^20^

We hypothesized that nodding syndrome is a neuroinflammatory disorder associated with *O volvulus* infection and systemic changes in immune and complement activation. To evaluate this, we measured markers of immune and complement activation in the cerebrospinal fluid and plasma of Ugandan children and adolescents with chronic nodding syndrome on anti‐epileptic drug therapy.

## 2 METHODS

### 2.1 Design

This was an exploratory case‐control study of neuroinflammation and peripheral inflammation in children and adolescents with a long‐standing diagnosis of nodding syndrome compared to frequency‐ and age‐matched controls. Cerebrospinal fluid (CSF) and plasma concentrations of a selected panel of cytokines, chemokines, and complement factors were assayed.

### 2.2 Setting

**FIGURE 1:**
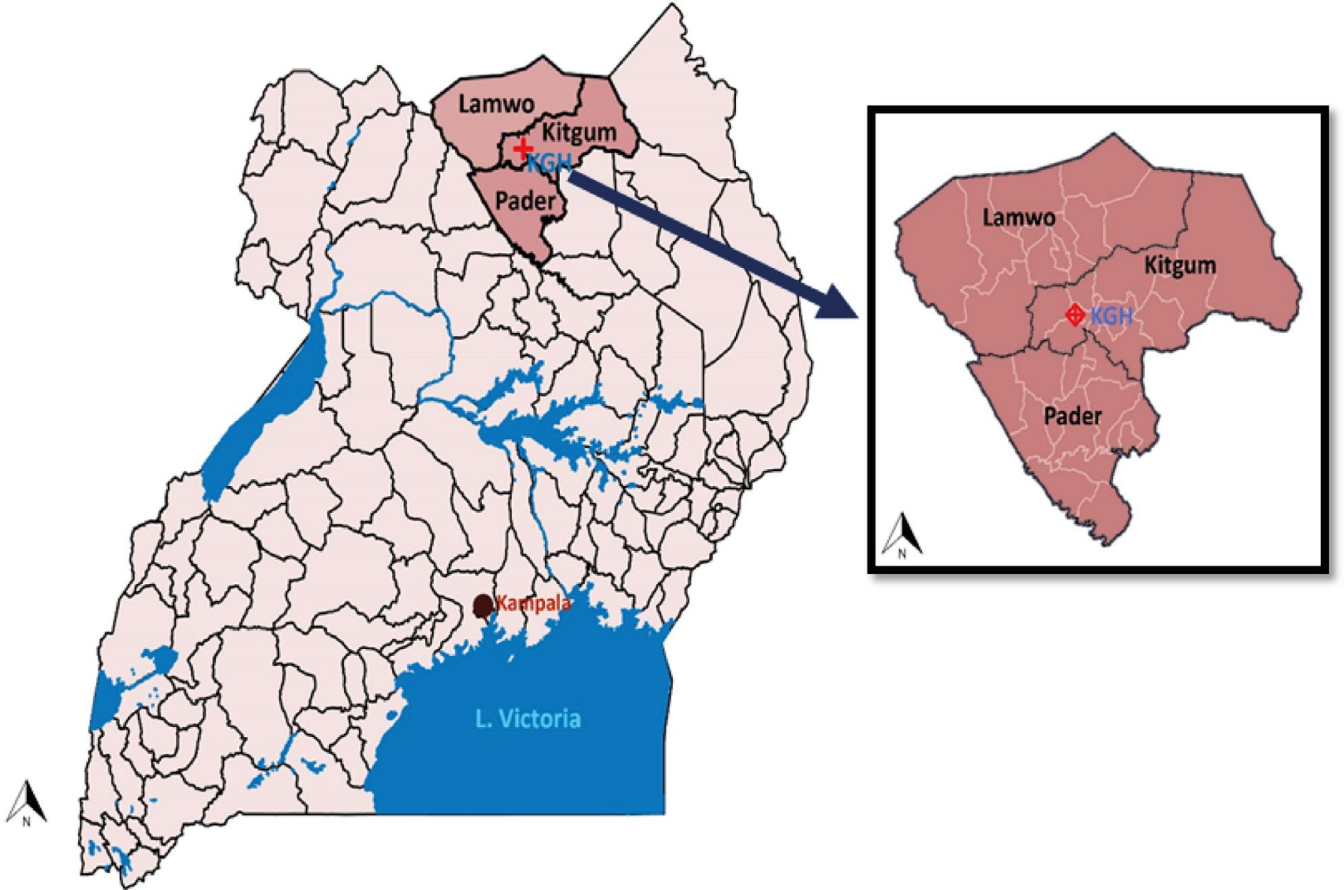
Study area in northern Uganda

The study was conducted in the nodding syndrome–affected districts of Kitgum, Pader, and Lamwo in northern Uganda (Figure 1). This region suffers high levels of poverty and is still recovering from a two‐decade‐old Lord's Resistance Army war against the Government of Uganda. Subsistence farming is the major economic activity. The age‐specific prevalence of nodding syndrome in the affected age‐group in these districts was approximately 6.8 (95% CI: 5.9‐7.7) per 1000.^4^ Study participants were screened for eligibility at the nodding syndrome treatment centers within the respective districts.^13^ Those enrolled were hospitalized in Kitgum General Hospital (KGH) where all study procedures were conducted. KGH is the largest epilepsy and nodding syndrome treatment center in the region.

### 2.3 Ethical approval

Ethical approval was obtained from Makerere University School of Medicine Research and Ethics Committee (SOMREC) and University of Oxford Tropical Medicine Research Ethics Committee (OXTREC). Regulatory approvals were provided by Uganda National Council for Science and Technology (UNCST). Written informed consent was obtained from the primary caregiver of all participants enrolled in the study. In the event, a caregiver was unable to read and write, consent was obtained using an independent witness, and the caregiver provided a fingerprint. Assent was obtained from all children, except from those that were severely cognitively impaired as judged by a trained psychologist. All consent forms are stored at the Centre of Tropical Neurosciences (CTN) for future reference.

### 2.4 Participants

The study recruited 154 children and adolescents with nodding syndrome receiving standard symptomatic care at the dedicated treatment centers and 154 healthy community controls from neighboring homes in the same communities. All nodding syndrome cases were 8 years or older, and the diagnosis was according to WHO criteria^21^: head nodding on two or more occasions, occurring in clusters at a frequency of 5‐20/minute; observed by a trained health worker; onset between 3 and 18 years; and plus any one of the following: a) triggered by food or cold weather; b) presence of other seizures or neurological abnormalities and cognitive decline; and c) clustering in space or time. The community controls were frequency‐matched based on prior defined age‐groups of 6‐9, 10‐14, and 15‐18 years. Control CSF samples were from fifteen Ugandan children in remission from a hematological malignancy as part of their routine follow‐up at the Uganda Cancer Institute in Mulago Hospital located in Kampala, Uganda.

### 2.5 Clinical history and examination

On enrollment, all participants had a detailed history taken to characterize the burden and type of seizures experienced in the previous month and a detailed physical examination including evaluations for neurologic, cognitive, and psychiatric disorders. Finally, following standard aseptic collection methods, EDTA‐anticoagulated blood and CSF by lumbar puncture were obtained. Plasma was separated by centrifugation, and all samples were stored at −20°C prior to transfer on dry ice to Kampala for long‐term storage at −80°C until testing. All samples were shipped on dry ice, and sample integrity was confirmed on arrival. Exposure to *O volvulus* was measured at the laboratory of parasitic research at the National Institute of Health, by evaluating levels of anti–OV‐16 IgG in plasma as previously described.^22^ A sample was considered seropositive for *O volvulus* when anti–OV‐16 IgG signal‐to‐noise ratio was above a cutoff of 2. The cutoff was determined previously using receiver operator characteristic curves using “other helminth” control samples.^22^ The presence of active *O volvulus* infection was determined by microscopy of saline solutions in which skin snips obtained from the iliac crest of all participants were incubated following standard microscopy protocols.

As part of national onchocerciasis control programs, all children in the region receive biannual ivermectin at a standard dose of 150 ug/kg. All nodding syndrome participants also received routine care including nutritional supplementation and physical therapy as necessary for rehabilitation, and symptomatic treatment of seizures with sodium valproate (dose rationalized between 10 and 35 mg/kg/day depending on seizure frequency).

A validated disease staging system was adapted to classify nodding syndrome participants into 3 categories: mild, moderate, and severe disease.^2^, ^7^ Cases were defined to have mild disease if they only had head nodding in the absence of convulsive seizures (tonic, clonic, myoclonic, absence, tonic‐clonic) or other disabilities. Moderately severe disease included children with head nodding and convulsive seizures: tonic, clonic, tonic‐clonic, myoclonic, or absence. Severe disease included cases with convulsive seizures and i) focal neurological deficits (eg, impaired motor function); ii) recognizable cognitive impairment, behavioral disorders, or psychiatric disorders; iii) severe malnutrition (severe stunting [height‐for‐age z score < −3] or severe wasting [BMI‐for‐age z score< −3]); iv) physical deformities including kyphosis, limb, and pectus deformities; and v) severe disability with limited independent mobility.

### 2.6 Inflammatory marker quantification

Markers of immune and complement activation were measured in plasma and CSF by enzyme‐linked immunoassays (ELISAs) or custom MAGPIX Luminex assay. Markers were selected based on previous associations with neuroinflammation in other neurological disorders.^23^, ^24^ Markers measured by ELISA were as follows: C‐reactive protein (CRP, dilution: 1:20,000 plasma, undiluted CSF), matrix metallopeptidase‐9 (MMP‐9, dilution: 1:2 plasma, undiluted CSF), and Chemokine (C‐C motif) ligand 5 (regulated on activation, normal T cell expressed and secreted [RANTES], dilution: 1:2 plasma, undiluted CSF) using commercially available kits (DuoSet^®^ ELISA Development System; R&D Systems, Minneapolis, MN, USA). Plasma was tested in duplicate, and CSF was tested in single replicate due to limited sample volume. Assays were performed according to the manufacturer's instructions except for overnight sample incubation at 4°C to increase assay sensitivity. Neopterin assays were performed using a competitive ELISA (DRG Instruments, GmbH, Germany) following the manufacturer's instructions. A custom‐developed multiplexed fluorescent magnetic bead–based immunoassay (R&D Systems, Minneapolis, USA) was used to measure the remaining markers (CXCL10, CXCL13, CXCL9, CCL2, APRIL, BAFF, IL‐6, IL‐4, IL‐13, IL‐10, TNF‐α, INF‐γ, C5/C5a) according to the manufacturer's instructions. Plasma samples were tested using a twofold dilution, and CSF samples were tested undiluted.

### 2.7 Statistical Methods

Epi Info™ version 7.1.5, R studio version 3.6.3, and GraphPad Prism version 6.05 for windows were used. Proportions were compared using Pearson's chi‐square test. For continuous data, the means of normally distributed data were compared using Student's t test as appropriate. In case this was not normally distributed, data were analyzed using the Mann‐Whitney U test and presented as medians (interquartile range, IQR). The relationship between markers of immune activation in CSF and seizure burden was evaluated using a Spearman rank correlation test. The normal range for CSF markers was estimated using the 95th centile of control values to establish the upper and lower limits of normal range. A multivariable logistic regression analysis was performed to evaluate plasma inflammatory markers associated with nodding syndrome adjusting for demographics. As *P* *falciparum* is associated with systemic inflammatory responses and there were differences in the prevalence of *P* *falciparum* infections between groups, the analysis was stratified by *P falciparum* status. Multicollinearity in models was assessed using the variance inflation factor (VIF) and confirmed that it was < 10.

## 3 RESULTS

### 3.1 General characteristics

The mean age of the population was 15.1 (SD 1.9) years, and 45.1% were female. The age and sex distribution between groups was similar (Table 1). The geographic location of the nodding syndrome cases and community controls is shown in Figure 1. The CSF controls were enrolled from Kampala in central Uganda. In children with nodding syndrome, the average time from nodding syndrome diagnosis to enrollment in the study was 8.3 (SD 2.7) years, and 62/154 (40.3%) of participants were seizure‐free in the previous 30 days. The major other seizure type reported was mainly generalized tonic‐clonic seizures, reported in 83/154 (53.8%) cases (Table 1). A total of 31/154 (20.1%) had mild, 34/154 (22.1%) had moderately severe, and 89/154 (57.8%) had severe disease (Table S1). The mean age was 15.5 (SD: 1.7), and 85/154 (55.2%) of the cases were male. A total of 9/154 (5.8%) nodding syndrome cases were severely stunted compared with 6/154 (3.9%) community controls, *P* = .59, while 35/154 (22.7%) cases were severely wasted compared with 17/154 (11.0%) community controls, *P* = .009.

**TABLE 1:** Study participant demographics and infections

Variable	Nodding syndrome (n=154)	Community controls (n=154)	CSF controls (n=15)	*P* value
Demographics				
Age, years, mean (SD)	15.5 (1.7)	14.6 (2.1)	12.3 (1.5)	<.0001^a^
Male, % (n/N)	55.2% (85/154)	54.5% (84/154)	46.7% (7/15)	.81^b^
Height‐for‐age z scores, median (IQR)	‐1.11 (1.81‐0.36)	‐0.8 (‐1.62‐0.01)	NA	.01^c^
Severe stunting, % (n/N) (z score<‐3)	5.8% (9/154)	3.9% (6/154)	NA	.59^b^
BMI‐for‐age z scores mean (SD)	‐2.02 (1.47)	1.65 (1.15)	NA	.01^c^
Severe wasting, % (n/N) (z score<‐3)	22.7% (35/154)	11% (17/154)	NA	.009^b^
MUAC, cm, mean (SD)	22.16 (3.33)	21.76 (2.84)	NA	.26^c^
Infection				
Malaria smear positive, % (n/N)	72.7% (112/154)	54.5% (84/154)	0% (0/15)	<.0001^b^
HIV infection, % (n/N)	0.6% (1/154)	0% (0/154)	0% (0/154)	.14^b^
Seropositive OV‐16, % (n/N)	95.4% (147/154)	55.2% (86/154)	NA	<.0001^b^
Active *O. volvulus* infection, % (n/N)	8.4% (13/154)	1.3% (2/154)	NA	.006^b^

Abbreviation: NA, data not available/applicable.

^a^ ANOVA.

^b^ Chi‐square test.

^c^ Mann‐Whitney U test.

^d^ Unpaired Student’s t test.

**FIGURE 2:**
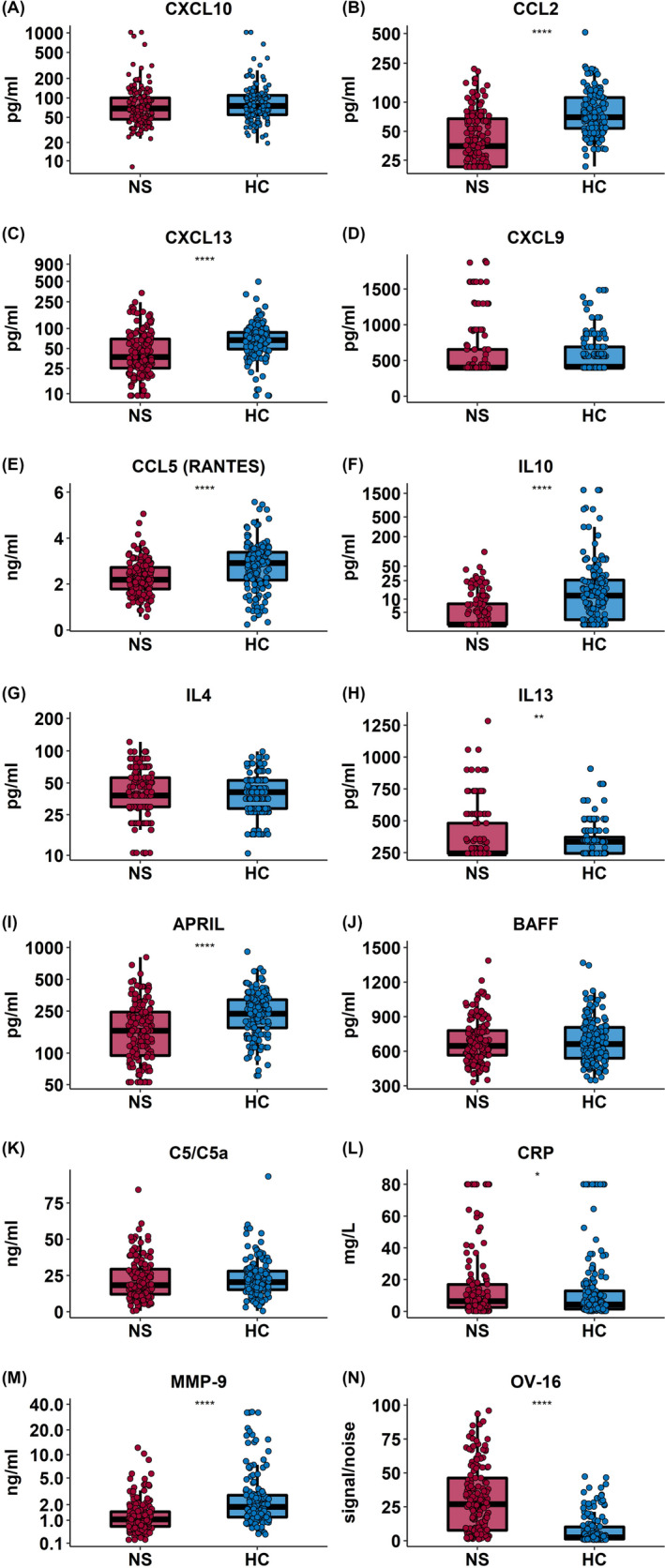
Plasma markers of immune activation in nodding syndrome and community controls. Box plots showing plasma levels of markers of immune activation in cases with nodding syndrome (nodding syndrome, n = 154, red) and healthy community controls (HC, n = 154, blue). Box plots represent median and IQR with whiskers denoting the 5% and 95% percentile data compared using the Mann‐Whitney U test, where *P ≤ .01, **P ≤ .001, ***P ≤ .0001, and ****P ≤ .00001. After adjusting for multiple comparisons, IL‐10, April, RANTES, CCL2, CXCL13, MMP‐9, and OV‐16 remained significant

Although none of the children were acutely ill at the time of enrollment, asymptomatic malaria was commonly occurring in 72.7% (112/154) of participants with nodding syndrome and 54.5% (84/154) of community controls with an odds ratio (OR) of 2.2 (95% CI: 1.34, 3.68, *P* = .001). Among children with nodding syndrome, there were no differences in the prevalence of malaria among children with mild, moderate, or severe disease (*p* trend = 0.88). One child with nodding syndrome was HIV‐infected, while none of the community children or cancer survivors tested positive for HIV infection. The majority of cases of nodding syndrome were seropositive for OV (147/154 (95.4%)) compared with community controls (86/154 (55.8%)), with an OR of 17.04 (95% CI: 7.33, 45.58, *P* < .0001). In addition, the cases had higher relative levels of anti–OV‐16 IgG, compared with the community controls, and the median signal‐to‐noise (S/N) ratio was 27.0 (IQR, 7.6‐46.3) in cases compared with 2.40 (IQR, 1.2‐10.3) in community controls (*P* < .0001) (Figure 2). There was a trend to an increased level of anti–OV‐16 IgG with disease severity with increasing median [IQR] across mild (12.07 [3.45‐49.31]), moderate (26.6 [9.4‐26.6]), and severe (31.4 [9.5‐48.9]) signal‐to‐noise ratio disease although this was not significant (*p* trend = 0.06). By skin snip microscopy, active *O volvulus* infection was observed in 13/154 (8.4%) cases (the microfilaria [MF] load ranged from 1 to 88 MF per skin snip) and only 2/154 (1.3%) among community controls (MF load ranged from 1 to 16 per skin snip). Children with nodding syndrome had increased odds of active *O volvulus* infection with an OR of 7.0 (95% CI: 1.53, 64.70, *P* = .006) compared to community controls.

### 3.2 Immune and complement activation in CSF of nodding syndrome

**FIGURE 3:**
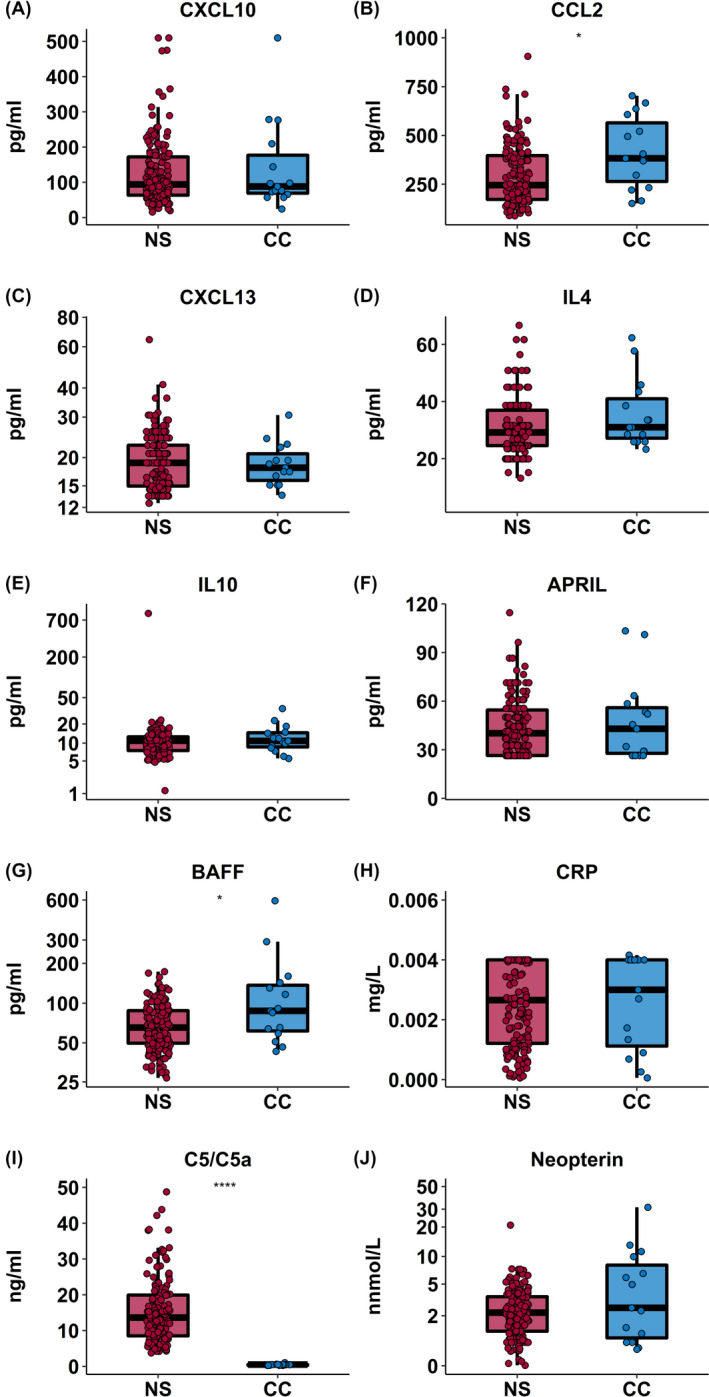
Cerebral spinal fluid inflammatory markers in patients with nodding syndrome. Box plots showing CSF levels of markers of immune activation in cases of nodding syndrome (NS, n = 154, red) compared with CSF controls (CC, n = 15, blue). Box plots represent median and IQR with whiskers denoting the 5% and 95% percentiles data compared using the Mann‐Whitney U test, where *P ≤ .01, **P ≤ .001, ***P ≤ .0001, and ****P ≤ .00001. After adjusting for multiple for multiple comparisons, only C5/C5a remained significant

To investigate the pathways of central nervous system inflammation in nodding syndrome, the levels of immune and complement activation were measured in CSF of children with nodding syndrome and CSF controls (Ugandan children in remission for an hematological malignancy). Compared to the normal range established in the CSF of CSF controls recovered from hematological malignancy, complement factor 5 (C5/C5a) was elevated in all cases of nodding syndrome compared with CSF controls (*P* < .0001) (Figure 3, Table S2). C‐reactive protein (CRP), another marker of complement activation, was elevated in 56 (36.3%) of cases relative to the population normal derived from CSF controls; however, there was no significant difference in CRP levels between cases and controls with an OR of 1.44 (95% CI: 0.38, 5.32) (Figure 3, Table 2). None of the other markers of immune activation were above or below the normal limits determined in greater than 5% of nodding syndrome cases (Table S3), and the levels were comparable between groups when adjusting for multiple comparisons. The levels of CXCL9, CCL5 (RANTES), IL‐13, IL‐6, TNF‐*α*, MMP‐9, and INF‐γ were undetectable in both cases and controls (Figure 3).

**TABLE 2:** Table comparing markers of immune activation in nodding syndrome case with mild, moderate, and severe disease

Marker	Plasma				CSF			
	Mild disease (n = 31)	Moderate disease (n = 34)	Severe disease (n = 89)	*P* trend	Mild disease (n = 31)	Moderate disease (n = 34)	Severe disease (n = 89)	*P* trend
Immune activation								
CXCL10, pg/mL	68.32 (50.59, 121.2)	64.85 (44.25, 86.81)	71.47 (43.5, 106.7)	.84	112.0 (64.4, 172.70)	103.6 (78.96, 214.7)	82.22 (55.06, 143.6)	.08
CCL2, pg/mL	38.28 (22.01, 63.76)	37.72 (21.24, 80.45)	34.56 (21.24, 63.76)	.42	256.3 (221.1, 396.1)	244.2 (176.1, 459.4)	237.5 (166.9, 390.7)	.36
CXCL13, pg/mL	44.73 (24.31, 70.09)	47.31 (25.29, 87.5)	34.8 (25.13, 66.83	.73	18.96 (15.43, 24.36)	18.96 (15.43, 23.5)	18.96 (14.48, 22.64)	.50
CXCL9, pg/mL	399.7 (399.7, 878.5)	399.7 (399.7, 923.3)	399.7 (399.7, 619.5)	.32	ND	ND	ND	‐
CCL5(RANTES) ng/mL	2.13 (1.94, 2.59)	2.19 (1.80, 2.62)	2.24 (1.60, 2.82)	.88	ND	ND	ND	‐
IL‐10, pg/mL	38.18 (29.8, 59.98)	41.25 (33.99, 56.33)	38.18 (20.79, 58.77)	.14	29.17 (24.59, 33.14)	26.89 (24.05, 38.65)	29.17 (24.39, 38.35)	.19
IL‐4, pg/mL	2.42 (2.42, 4.93)	2.42 (2.42, 4.63)	2.42 (2.42, 9.87)	.21	11.27 (8.02, 13.68)	11.38 (8.54, 13.64)	9.65 (7.23, 12.17)	.99
IL‐13, pg/mL	245.2 (245.2, 554.2)	245.2 (245.2, 554.2)	245.2 (245.2, 357)	.36	ND	ND	ND	‐
APRIL, pg/ml	149.7 (97.65, 256.8)	184 (110.7, 267.3)	163.3 (91.48, 230.9)	.87	45.66 (32.64, 60.87)	41.29 (26.45, 53.42)	36.99 (26.43, 50.08)	.06
BAFF, pg/mL	664.0 (570.1, 891.9)	643.8 (553.4, 852.9)	647.5 (568.5, 769.5)	.71	73.65 (58.85, 96.54)	63.41 (49.72, 92.05)	59.58 (47.98, 85.4)	.07
MMP‐9, ng/mL	0.87 (0.65, 1.53)	1.16 (0.74, 1.52)	1.05 (0.71, 1.50)	.71	ND	ND	ND	‐
Neopterin, mmol/L	Not measured	Not measured	Not measured	‐	2.268 (1.23, 3.90)	2.715 (0.95, 4)	2.84(1.43, 4.0)	.53
Complement activation								
C5/C5a, ng/mL	18.06 (11.54, 29.87)	23.45 (16.69, 31.51)	16.89 (11.33 29.50)	.47	14.90 (9.53, 21.31)	14.68 (8.73, 18.96)	11.99 (7.79, 19.37)	.14
CRP, mg/L	4.11 (0.88 ‐ 8.26)	5.58 (2.15, 22.48)	7.71 (4.02, 17.59)	.008	0.0029 (0.001, 0.003)	0.0024 (0.001, 0.003)	0.00214 (0.0008, 0.003)	0.33

Note

Data presented as median (IQR) and analyzed using a nonparametric test for trend.

Abbreviations: April, A proliferation‐inducing ligand; BAFF, B lymphocyte stimulator; C5/C5a, complement component; CCL2, chemokine (C‐C motif) ligand 2; CCL5 (RANTES), chemokine (C‐C motif) ligand 5 (regulated on activation, normal T cell expressed and secreted); CRP, C‐reactive protein; CSF, cerebral spinal fluid; CXCL10, C‐X‐C motif chemokine 10/ interferon gamma‐induced protein 10; CXCL13, chemokine (C‐X‐C motif) ligand 13; CXCL9, chemokine (C‐X‐C motif) ligand 9; IL‐10, interleukin‐10; IL‐13, interleukin‐13; IL‐4, interleukin‐4; MMP‐9, matrix metallopeptidase 9; ND, not detected.

Next, we evaluated whether there were differences in the median levels of CSF markers of immune and complement activation in nodding syndrome cases by disease severity (Table 2). No differences in markers of immune or complement activation by disease severity were observed according to analysis using a nonparametric test for trend. Among nodding syndrome cases, there was no correlation between markers of immune or complement activation and malaria, seizure burden, dose of sodium valproate (mg/kg/day), OV‐16 exposure (S/N), and duration of disease (years) (Figure S1).

### 3.3 Systemic immune activation in nodding syndrome cases compared with community controls

To investigate whether nodding syndrome is associated with systemic changes in inflammatory responses, plasma levels of markers of immune or complement activation were measured in nodding syndrome cases compared with healthy community controls. In plasma, using the clinically recognized cutoff for elevated CRP levels (≥ 10 mg/L), CRP was elevated in 55/154 (33.7%) cases compared with 48/154 (31.1%) controls (*P* = .46). The median CRP levels were 6.45 (2.43‐12.19) mg/L in cases compared with 4.34 (1.45‐13.35) mg/L in the healthy controls, with an OR of 1.22 (95% CI: 0.83, 1.80). Among nodding syndrome cases, there was a stepwise increase in median CRP levels with disease severity (*p* trend = 0.008) (Table 2). Furthermore, CSF and plasma CRP levels were strongly correlated (r = 0.7, *P* > .0001) (Figure S2).

**FIGURE 4:**
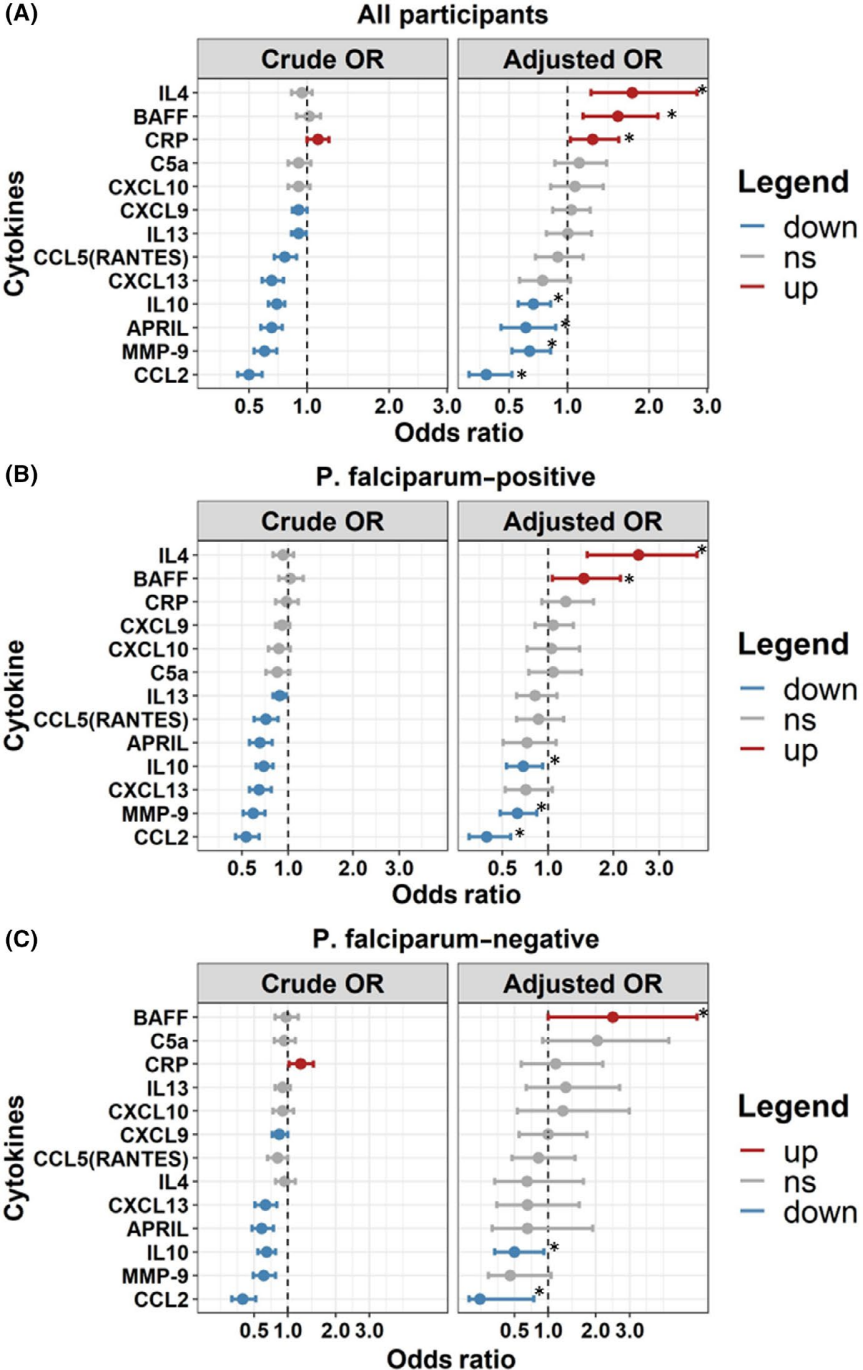
Forest plot depicting the relationship between plasma markers of inflammation in children with nodding syndrome and community children. Forest plots show odds ratio (95% CI) from a multivariable logistic regression model based on a one‐unit change in log 10‐transformed plasma biomarker with nodding syndrome cases as the dependent variable. (A) Model with all participants (NS = 154 and CC = 154). (B) Model in *P falciparum*–positive individuals (NS = 112, CC = 84). (C) Model in *P falciparum*–negative participants (NS = 42, CC = 70). Adjusted models included participant age, sex, BMI‐for‐age z score, *O volvulus* seropositivity, and *P falciparum* status (in combined model). Plasma markers upregulated in nodding syndrome are shown in red, markers with no change are shown in gray, and markers downregulated are depicted in blue. Markers significant following adjustment for multiple comparisons are indicated with an asterisk. Markers are ordered by odds ratio from highest to lowest within each plot

Inflammatory markers IL‐10, APRIL, CCL5 (RANTES), CCL2, CXCL13, and MMP‐9 were significantly lower among cases compared with community controls after adjusting for multiple comparisons. There was no significant difference in the level of C5/C5a, IL‐4, BAFF, or CXCL9 between cases and community controls (Figure 2). In multivariable models, elevated levels of CRP, BAFF, and IL‐4 were independently associated with nodding syndrome, while CCL2, IL‐10, APRIL, and MMP‐9 were lower in children with nodding syndrome. When stratifying by *P falciparum* infection, elevated BAFF and downregulated CCL2 and IL‐10 were independently associated with nodding syndrome in children who were malaria‐positive and malaria‐negative (Figure 4).

Among nodding syndrome cases, there was no correlation between plasma markers of immune or complement activation and seizure burden, dose of sodium valproate (mg/kg/day), OV‐16 exposure (S/N), and duration of illness (years) (Figure S1). Furthermore, cytokine levels were not significantly affected by *O volvulus* infection or nutritional status (Tables S4, S5).

## 4 DISCUSSION

This study aimed to examine pathways of immune and complement activation in children with chronic nodding syndrome. We observed evidence of complement system activation in the CSF of children with nodding syndrome with elevated levels of CRP and C5/C5a. The findings suggest a possible role of central nervous system complement activation in the pathogenesis of chronic nodding syndrome. There was evidence of altered immune activation in children with chronic nodding syndrome with several markers of immune activation lower in children with nodding syndrome compared to community children. Further investigation of the complement system and immune dysregulation is required, particularly in children with acute nodding syndrome.

Inflammation is associated with epileptic and neurological disorders and has been shown to exacerbate disease progression.^25^, ^26^ In the context of nodding syndrome, a recent postmortem study identified gliosis and features of past ventriculitis and/or meningitis in 80% of cases studied suggesting nodding syndrome maybe a postinfectious encephalopathy or autoimmune disorder.^20^ Although autoreactive antibodies have been observed in independent case‐control studies, no conclusive evidence of neuroinflammation has been demonstrated. In the present study, despite elevated CRP levels in CSF of one‐third of cases and a significant elevation of complement factor C5/C5a in CSF, there is limited evidence of immune activation in both the CSF and peripheral circulation of the cytokines and chemokines tested. Regardless, the observation of elevated CRP and that of complement activation in CSF are suggestive of ongoing innate immune responses within the CSF of children with nodding syndrome. Additional studies to understand pathways of complement activation and regulation in samples from children with both acute and chronic nodding syndromes, and comparison with CSF from children with other chronic neurodegenerative disorders may help elucidate the role of the complement system in nodding syndrome pathogenesis.

It is unclear why the levels of APRIL, IL‐10, IL‐13, CXCL10, CXCL13, and CCL2 were reduced in plasma of children with nodding syndrome compared to controls, but similar decreases in the levels of Th 2 type responses have been observed in individuals with prolonged occult *O volvulus* infection.^27^ Furthermore, children affected with nodding syndrome are more likely to have a history of chronic malnutrition or other infections, which may further alter their immune profiles. Interestingly, in all multivariable models nodding syndrome was associated with higher plasma levels of BAFF, even after stratifying for malaria infection, which is consistent with observations in autoimmune disorders such as systemic lupus erythematosus (SLE)^28^ and Sjögren's syndrome.^29^ These results support an immunological component to the pathophysiology of nodding syndrome.

The mean duration of nodding syndrome in this study was 8.3 years. During this timeframe, children were treated using a standard treatment plan aimed at symptomatic care.^30^ This included long‐term use of the anti‐epileptic sodium valproate for seizure control.^21^ Sodium valproate is documented to have anti‐inflammatory properties and may have further modulated immune responses.^31^, ^32^, ^33^ These may explain the absence of significant immune dysregulation among children receiving treatment.^30^ Furthermore, all children in the region received ivermectin biannually, which has been shown to transiently impact cytokine levels.^34^ These may have affected the level of immune activation leading to normalization of responses over time. Finally, the region is endemic to several infectious diseases, which may have confounded inflammatory levels in both cases and controls.^35^, ^36^, ^37^ Although the study examined the effect of malaria, the present study did not systematically evaluate other infections, including helminths that have immunomodulatory properties.^38^

To the best of our knowledge, this is the first study to demonstrate an association between complement activation and nodding syndrome. Several cells of the nervous system (astrocytes, neurons, endothelial cells, and oligodendrocytes) express complement proteins and receptors.^39^ Complement activation has been well‐described in other neurological conditions, including epilepsy (eg, postviral epilepsy, CASPR2‐associated encephalitis, and Rasmussen's syndrome),^40^, ^41^ major psychiatric disorders (eg, major depressive disorder and schizophrenia),^42^ and neurological disorders (eg, Alzheimer's disease, amyotrophic lateral sclerosis, Huntington's disease, and parkinsonism).^43^ Complement‐associated proteins have been implicated in several neurodegenerative disorders, and C5/C5a has been associated with neuronal death in vitro.^43^, ^44^ In the present study, peripheral elevation of complement was not observed suggesting interictal synthesis of C5a may occur in nodding syndrome as reported in the Guillain‐Barré syndrome.^45^ Multiple causes of complement dysregulation have been demonstrated including auto‐antibodies, damaged cells, exposed myelin antigens, pathogens, trauma, or anoxia.^39^, ^40^, ^43^ The precise mechanism by which this dysregulation may occur or result in nodding syndrome disease pathology is unclear. We speculate that complement activation may be secondary to an underlying etiological factor resulting in interictal complement synthesis.

It has been suggested that nodding syndrome may be a postviral disorder analogous to measles‐associated SSPE based on clinical and neuropathological overlaps.^18^ Therefore, in a similar fashion to viral encephalitis,^46^ and septic encephalopathy,^47^ the invasion of CSF by a neurotropic viral infection may trigger complement activation. In line with this observation, early evidence demonstrated complement activation in measles infections.^48^ Nodding syndrome has been liked strongly to *O volvulus;* however, the invasion of CSF by *O volvulus* has not been demonstrated. Despite this, it is possible that CSF invasion by onchocerca metabolites may occur resulting in immune and/or complement activation. *O volvulus* has been shown to activate the complement cascade; however, it can also bind human complement factor H, which regulates complement activation by cleaving C3b into an inactive form averting continued activation.^27^ A breakdown in this regulation within the CSF of children with nodding syndrome may have adverse effects. Finally, previously identified auto‐antibodies^13^, ^19^ may be neurotoxic through a complement antibody‐mediated mechanism.

The study had limitations. Children with nodding syndrome were assessed on average 8 years after their initial diagnosis and following treatment. Children had also been on medications for a number of years (including biannual treatment of *O volvulus* infection) that could modulate immune responses and affect conclusions drawn. Therefore, prospective enrollment of a cohort of children with new‐onset nodding syndrome will be important to extend and validate these findings. The CSF controls used in this study were Ugandan children in remission for an hematological malignancy. As it is unethical to collect CSF from healthy community children, we enrolled controls without evidence of infection or active disease undergoing lumbar puncture as part of follow‐up care for prior hematological malignancy. These patients may have had a substantial difference in nutritional status and history of infection compared to children with nodding syndrome. While we cannot definitively state that the levels of immune activation in the controls are representative of a healthy population, they are consistent with the levels reported in the literature.^23^

Strengths of this study include the enrollment of a large population of children with nodding syndrome and inclusion of controls from the same community. This population is well‐characterized, and data on seizure history, disease staging, and medication use were systematically collected, which enabled comparisons between pathways of interest and disease severity.

## 5 CONCLUSION

This study demonstrates evidence of complement activation in the cerebrospinal fluid of Ugandan children with nodding syndrome. However, in this population with chronic nodding syndrome, receiving symptomatic therapy of whom approximately two‐fifths had attained good seizure control, there was no evidence of systemic immune activation. Future studies are needed to evaluate the spectrum of complement proteins and inflammation in incident cases in order to design therapies that can be used in the acute phase of disease to mitigate disease progression and improve long‐term outcomes in children with nodding syndrome.

## CONFLICT OF INTEREST

None of the authors has any conflict of interest to disclose.

## ETHICAL PUBLICATION STATEMENT

We confirm that we have read the Journals position on issues involved in ethical publication and affirm that this report is consistent with those guidelines.

## Supporting information

Supplementary Material
