# Supplementary material for: Systemic and cerebrospinal fluid immune and complement activation in Ugandan children and adolescents with long‐standing nodding syndrome: A case‐control study
Source: Epilepsia Open. 2021 Mar 12;6(2):297–309. doi: 10.1002/epi4.12463 (PMC8166803; doi:10.1002/epi4.12463)
Supplement: Supplementary file 1 — Supplementary Material [file EPI4-6-297-s001.pdf]

Supplementary

Figure S1: Correlation matrix between inflammatory marker levels and clinical factors among children with nodding syndrome

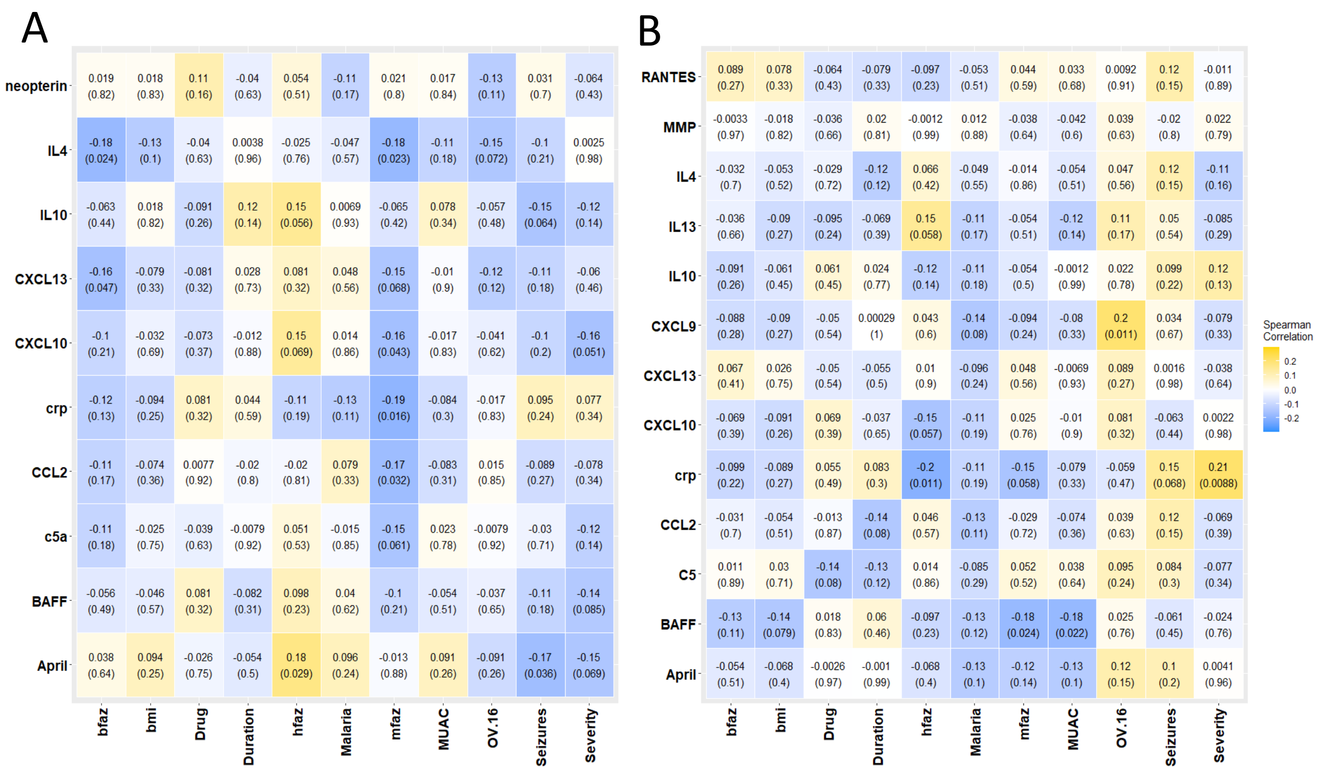

Correlation matrix between biomarker levels in CSF (A), plasma (B) and clinical factors. Matrices show the inflammatory biomarker level on the y-axis and selected clinical factors on the x-axis with the Spearman rho values and corresponding p-values (rho (p -value)) in each box. The matrices are coloured based on direction of correlation: gold is a positive correlation, while blue is a negative correlation. The clinical factors evaluated were: malaria (positive by blood smear), duration (number of years with disease), seizures (number of seizures in the last month), drug (dose in mg/kg/day of sodium valproate), bmi (body mass index), bfaz (bmi-for-age z score), hfaz (height-for-age z score), MUAC (mid upper arm circumference), mfaz (mid upper arm circumference for age z score, disease severity (disease stage mild, moderate, severe disease)

Figure S2: Correlation matrix between plasma and CSF marker levels cytokine among nodding syndrome participants.

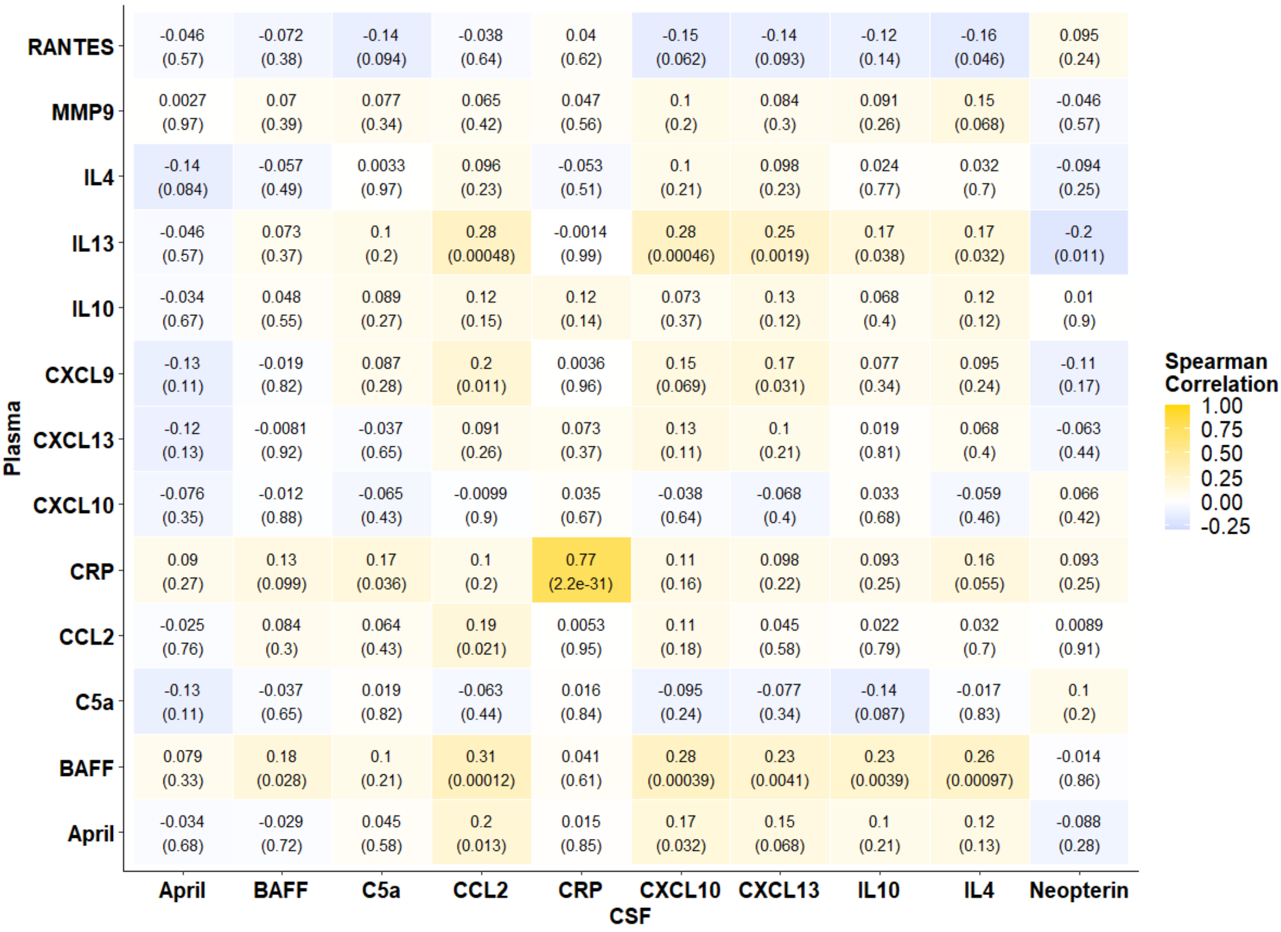

Correlation matrix between biomarker levels in CSF and plasma. Matrices show the plasma biomarker level on the y-axis and the CSF biomarkers on the x-axis with the Spearman rho values and corresponding p-values (rho (p -value)) in each box. The matrices are coloured based on direction of correlation: gold is a positive correlation, while blue is a negative correlation.

Table S1. Characteristics of children with nodding syndrome based on severity of illness

**Variable**	**Mild disease (n=31)**	**Moderate disease (n=34)**	**Severe disease (n=89)**	**P trend**
**Demographics**				
Age, years, median (IQR)	16 (15, 17)	15 (15, 16)	16 (15, 17)	0.59
Male, % (n/N)	64.5% (20/31)	50.0% (17/34)	53.9% (48/89)	0.62
Height for age z score	-0.8 (-2.0, -0.2)	-0.8 (-1.4, -0.2)	-1.3 (-1.9, -0.5)	0.059
Severe stunting, % (n/N) (z score<-3)	3.2% (1/31)	0.0% (0/34)	8.9% (8/89)	0.12
BMI for age z score	-1.9 (-2.3, -1.0)	-1.4 (-2.4, -1.0)	-1.9 (-3.4, -1.1)	0.14
Severe wasted, % (n/N) (z score<-3)	9.6% (3/31)	14.7% (5/34)	30.3% (27/89)	0.009
MUAC, cm, median (IQR)	23.2 (21.2, 25.0)	22.6 (21.2, 24.8)	21.2 (19.0, 23.5)	0.006
**History of Illness**				
Duration of illness, years	8 (5, 9)	9 (6, 10)	9 (7, 10)	0.01
Ongoing seizures, % (n/N)	0.0% (0/31)	79.4% (27/34)	73.0% (65/89)	<0.0001
Seizure burden, median (IQR)	2.0 (0, 4)	0.0 (0, 4)	2.0 (0, 4)	0.43
Dose sodium valproate, mg/kg	20.4 (16.8, 24.5)	20.4 (17.0, 24.4)	24.4 (19.7, 28.6)	0.01
**Infection Status**				
Malaria positive, % (n/N)	75.0% (24/31)	69.7% (23/34)	73.0% (65/89)	0.88
HIV infection, % (n/N)	0.0% (0/31)	3.0% (1/34)	0.0% (0/89)	0.42
Seropositive OV16 % (n/N)	90.0% (28/31)	97.0% (33/34)	95.5% (85/89)	0.18
Active *O. volvulus* infection, % (n/N)	0.0% (0/31)	0.0% (0/34)	14.6% (13/89)	0.003

Table S2. Comparing markers of immune activation in CSF of children with nodding syndrome and controls

	**Nodding syndrome cases N=154**	**CSF controls N=15**	**OR (95% CI)**	**P value**
**Immune activation**				
CXCL10, pg/ml	94.04 (63.08, 173.1)	82.46 (59.52, 193.30)	0.74 (0.12, 4.30)	0.74
CCL2, pg/ml	245.1 (171.9, 400.3)	380.6 (223.6, 585.9)	**0.04 (0.004, 0.443)**	0.008
CXCL13, pg/ml	18.96 (12.53, 22.64)	17.69 (15.13, 21.53)	1.96 (0.02, 179.41)	0.77
CXCL9, pg/ml	ND	ND	-	-
CCL5 (RANTES) ng/ml	ND	ND	-	-
IL10, pg/ml	10.86 (07.53, 12.6)	10.67 (07.63, 14.78)	0.33 (0.03, 3.41)	0.35
IL4, pg/ml	29.17 (24.59, 38.19)	31.0 (25.90, 42.21)	0.03 (0.00, 1.39)	0.07
IL13, pg/ml	ND	ND	-	-
APRIL, pg/ml	40.21 (26.44, 54.78)	37.50 (26.43, 57.19)	0.38 (0.02, 9.89)	0.57
BAFF, pg/ml	66.48 (49.72, 88.78)	87.51 (59.11, 143.4)	**0.97 (0.96, 0.98)**	0.001
MMP-9, ng/ml	ND	ND	-	-
Neopterin, mmol/L	2.39 (1.35, 3.57)	3.75 (0.76, 9.11)	0.36 (0.10, 1.25)	0.10
**Complement activation**				
C5/C5a, ng/ml	11.82 (7.518, 16.53)	0.49 (0.31, 0.551)	#	≤0.0001
CRP**,** mg/ml	2.52 (1.06, 4.00)	4.00 (0.90, 4.00)	1.44(0.38, 5.32)	0.58

Data presented as median (IQR)
Odds ratio (OR) and 95% confidence interval (CI) calculated using logistic regression where expressed for a one-unit increase in log10 transformed biomarker levels
Abbreviations: CXCL10 – C-X-C motif chemokine 10/ Interferon gamma-induced protein 10, CCL2 – chemokine (C-C motif) ligand 2, CXCL13 – chemokine (C-X-C motif) ligand 13, CXCL9 – Chemokine (C-X-C motif) ligand 9, CCL5(RANTES) – Chemokine (C-C motif) Ligand 5 (regulated on activation, normal T cell expressed and secreted), IL10 – Interleukin 10, IL4 – Interleukin 4, IL13 – Interleukin 13, April – A proliferation-inducing ligand, BAFF – B lymphocyte stimulator, MMP-9 – Matrix metallopeptidase 9, CRP – C -reactive protein, C5/C5a – Complement component, CSF – Cerebral spinal fluid , ND – Not detected , # Logistic estimate logC5a > 3.02 predicts data perfectly.

Table S3. Table of showing the frequency of children with nodding syndrome with elevated markers of CSF immune activation.

Marker	5th centile (lower limit CSF controls)	95th centile (upper limit CSF controls)	Number (%) of nodding syndrome cases with elevated marker levels
CXCL10, pg/ml	5.41	510	3 (0%)
CCL2, pg/ml	10.62	704	3 (1.94%)
CXCL13, pg/ml	4.65	30.61	5 (4.54%)
IL4, pg/ml	7.69	62.38	1 (0.64%)
IL10, pg/ml	1.21	34.26	1 (0.64%)
APRIL, pg/ml	26.43	103.4	1 (1.29%)
BAFF, pg/ml	22.75	592.3	0 (0%)
CRP, mg/L	0.00006	0.004	56 (36.3%)
C5/C5a, ng/ml	0.280	1.04	154 (100.0%)
Neopterin, nmol/L	0.44	31.4	0 (0%)

The 5th and 95th centile represent the biomarker cut-offs based on the healthy Ugandan CSF controls. Abbreviations: CXCL10 – C-X-C motif chemokine 10/ Interferon gamma-induced protein 10, CCL2 – chemokine (C-C motif) ligand 2, CXCL13 – chemokine (C-X-C motif) ligand 13, IL4 – Interleukin 4, IL10 – Interleukin 10, April – A proliferation-inducing ligand, BAFF – B lymphocyte stimulator, CRP – C -reactive protein, C5/C5a – Complement component 5a.

Table S4. Differences in immune activation based on *O. volvulus* infection

	Nodding Syndrome				Community Children			
**Markers**	**Positive N=147**	**Negative N=7**	**OR (95% CI)**	**P value**	**Positive N=86**	**Negative N=68**	**OR (95% CI)**	**P value**
**Immune activation**								
CXCL10, pg/ml	70.15 (46.77, 102.24)	49.54 (33.7, 76.14)	6.96 (0.35, 135.23)	0.17	73.05 (50.46, 101.65)	87.87 (56.19, 115.47)	0.34 (0.10, 1.17)	0.13
CCL2, pg/ml	35.85 (21.24, 66.49)	29.97 (21.24, 110.83)	0.94 (0.06 ,14.60)	0.90	71.19 (53.79, 112.22)	68.71 (51.99, 111.65)	2.77 (0.577, 13.30)	0.19
CXCL13, pg/ml	40.88 (25.29, 71.05)	23.98 (17.87, 46.1)	12.35 (0.74,206,50)	0.10	66.25 (45.11, 91.29)	68.56 (49.85, 85.88)	1.56 (0.43, 5.59)	0.90
CXCL9, pg/ml	399.7 (399.70, 703.94)	399.7 (399.70, 403.70)	87.12 (0.04,1887.44)	0.36	560.18 (399.70, 689.88)	412.61 (399.70, 593.67)	2.39 (0.30, 18.75)	0.24
CCL5 (RANTES) ng/ml	2.17 (1.78, 2.74)	2.35 (1.72, 2.62)	0.42 (0.0015,114.4)	0.83	2.95 (2.05, 3.38)	2.87 (2.30, 3.39)	0.82 (0.18, 3.73)	0.57
IL10, pg/ml	2.42 (2.42, 8.04)	2.42 (2.42, 3.12)	21.16 (0.13, 3424)	0.25	9.68 (2.82, 25.51)	14.23 (6.02, 32.76)	0.73 (0.45, 1.16)	0.11
IL4, pg/ml	38.18 (29.80, 56.33)	29.80 (20.79, 56.33)	8.89 (0.35, 225.29)	0.30	35.66 (28.78, 53.82)	41.12 (28.78, 53.16)	1.32 (0.22, 7.64)	0.82
IL13, pg/ml	245.18 (245.18, 554.15)	245.18 (245.18, 357.02)	13.59 (0.06, 2939.77)	0.81	336.22 (245.18, 423.82)	246.64 (245.18, 347.36	5.42 (0.55, 53.63)	0.12
APRIL, pg/ml	163.27 (97.65, 246.24)	142.98 (67.74, 287.89)	2.19 (0.12, 39.08)	0.80	235.39 (175.31, 311.22)	239.70 (165.4, 333.61)	0.92 (0.19, 4.39)	0.78
BAFF, pg/ml	647.47 (567.31, 780.41)	589.44 (451.86, 951.64)	10.52(0.009, 11565)	0.59	642.90 (527.56, 766.84)	686.20 (542.19, 860.28)	0.13 (0.009, 2.03)	0.16
MMP-9, ng/ml	1.05 (0.70, 1.49)	1.23 (0.63, 1.41)	1.15 (0.08, 16.35)	0.99	1.85 (1.17, 3.21)	1.76 (1.20, 2.82)	1.60 (0.67, 3.80)	0.52
**Complement activation**								
C5/C5a, ng/ml	18.26 (12.04, 30.44)	18.62 (7.60 ,23.44)	1.87 (0.2, 17.02)	0.58	21.09 (14.86, 27.78)	19.81 (15.44, 29.44)	0.78 (0.23, 2.61)	0.92
CRP, mg/ml	6.33 (2.49, 16.70)	14.25 (1.41, 64.02)	0.64 (0.18, 2.28)	0.36	4.14 (1.42, 10.52)	4.49 (1.51, 19.06)	0.82 (0.49, 1.37)	0.19

Data presented as median (IQR)

Plasma odds ratio (OR) and 95% confidence interval (CI) calculated using logistic regression adjusting for age and sex where OR expressed for a one-unit increase in log10 transformed biomarker levels

Abbreviations: CXCL10 – C-X-C motif chemokine 10/ Interferon gamma-induced protein 10, CCL2 – chemokine (C-C motif) ligand 2, CXCL13 – chemokine (C-X-C motif) ligand 13, CXCL9 – Chemokine (C-X-C motif) ligand 9, CCL5(RANTES) – Chemokine (C-C motif) Ligand 5 (regulated on activation, normal T cell expressed and secreted), IL10 – Interleukin 10, IL4 – Interleukin 4, IL13 – Interleukin 13, APRIL – A proliferation-inducing ligand, BAFF – B lymphocyte stimulator, MMP-9 – Matrix metallopeptidase 9, CRP – C -reactive protein, C5/C5a – Complement component.

Table S5. Differences in immune activation in plasma based on nutritional status

	Stunted	Not stunted		Wasted	Not wasted	
**Nodding Syndrome**						
**Markers**	**N= 9**	**N=145**	**P value**	**N=35**	**N=119**	**P value**
CXCL10, pg/ml	90.9 (60.9, 141)	67.5 (46.7, 99.8)	0.25	76.1 (48.7, 127.4)	67.5 (45.4, 99.8)	0.20
CCL2, pg/ml	27.9 (23.7, 72.6)	35.9 (21.2, 66.5)	0.86	30.0 (21.2, 63.8)	36.8 (21.2, 68.3)	0.60
CXCL13, pg/ml	49.7 (29.5, 114.3)	36.1 (25.1, 67.2)	0.29	33.5 (20.9, 62.1)	41.2 (25.3, 72.3)	0.16
CXCL9, pg/ml	399.7 (399.7, 403.7)	399.7 (399.7, 655.2)	0.63	399.7 (399.7, 655.2)	399.7 (399.7, 850.1)	0.40
CCL5 (RANTES) ng/ml	2.50 (1.62, 2.81)	2.18 (1.81, 2.72)	0.68	1.82 (1.40, 2.64)	2.24 (1.94, 2.80)	0.01
IL10, pg/ml	2.42 (2.42, 12.09)	2.42 (2.42, 7.64)	0.95	5.04 (2.42, 15.25)	2.42 (2.42, 5.35)	0.007
IL4, pg/ml	29.8 (20.8, 70.9)	38.2 (29.8, 56.3)	0.73	38.2 (20.8, 56.3)	38.2 (29.8, 61.2)	0.81
IL13, pg/ml	245.2 (245.2, 333.6)	245.2 (245.2, 481.0)	0.64	245.2 (245.2, 333.6)	245.2 (245.2, 554.2)	0.44
APRIL, pg/ml	120.0 (116.7, 287.9)	163.3 (93.6, 246.2)	0.52	163.3 (91.5, 237.6)	163.3 (94.0, 246.2)	0.78
BAFF, pg/ml	646.1 (565.1, 810.8)	647.5 (567.8, 780.4)	0.73	688.7 (585.0, 825.2)	643.8 (554.1, 780.4)	0.38
MMP-9, ng/ml	0.88 (0.70, 1.13)	1.10 (0.71, 1.51)	0.27	0.95 (0.70, 1.47)	1.11 (0.70, 1.51)	0.42
C5/C5a, ng/ml	10.7 (8.2, 27.9)	18.6 (13.1, 29.7)	0.33	16.9 (10.6, 23.9)	18.8 (13.9, 30.5)	0.09
CRP, mg/ml	8.43 (2.43, 11.48)	6.33 (2.51, 17.08)	0.96	9.06 (3.58, 50.55)	6.33 (2.42, 12.29)	0.11
**Community Children**						
**Markers**	**N=6**	**N=148**	**P value**	**N=17**	**N=137**	**P value**
CXCL10, pg/ml	78.38 (53.82, 104.6)	75.13 (54.8, 110.1)	0.84	67.38 (55.82, 106.7)	76.05 (54.15, 110.5)	0.63
CCL2, pg/ml	65.20 (52.78, 116.2)	69.89 (53.78, 111.7)	0.95	74.39 (58.87, 108.1)	69.89 (51.99, 112.7)	0.78
CXCL13, pg/ml	75.68 (60.16, 104.9)	66.9 (47.0, 87.5)	0.31	73.01 (50.18, 98.64)	66.85 (48.34, 86.79)	0.92
CXCL9, pg/ml	503.1 (399.7, 689.9)	412.6 (399.7, 689.9)	0.65	560.2 (399.7, 689.9)	412.6 (399.7, 689.9)	0.86
CCL5 (RANTES) ng/ml	3.18 (2.17, 3.71)	2.92 (2.14, 3.38)	0.45	3.07 (2.13, 3.52)	2.92 (2.22, 3.38)	0.50
IL10, pg/ml	70.09 (3.06, 719.4)	11.96 (3.28, 24.70)	0.14	14.09 (4.27, 24.2)	11.96 (3.28, 26.2)	0.94
IL4, pg/ml	44.77 (38.04, 53.33)	38.39 (28.78, 53.16)	0.42	44.77 (32.22, 53.16)	41.12 (28.78, 53.16)	0.57
IL13, pg/ml	380.0 (245.2, 509.3)	336.2 (245.2, 347.4)	0.58	336.2 (245.2, 347.3)	336.2 (245.2, 423.8)	0.78
APRIL, pg/ml	238.1 (230.9, 329.2)	237.0 (169.6, 321.7)	0.76	285.7 (212.4, 379.6)	233.8 (173.9, 319.8)	0.22
BAFF, pg/ml	769.0 (678.2, 825.9)	650.0 (531.8, 815.8)	0.19	760.0 (652.8, 979.3)	635.2 (521.7, 796.4)	0.009
MMP-9, ng/ml	1.67 (1.53, 4.55)	1.85 (1.16, 2.85)	0.54	2.23 (1.55, 2.56)	1.18 (1.17, 2.87)	0.73
C5/C5a, ng/ml	29.95 (24.0, 45.2)	19.99 (15.15, 27.75)	0.08	23.96 (18.48, 28.50)	19.89 (15.00, 27.93)	0.42
CRP, mg/ml	4.87 (4.06, 12.89)	4.30 (1.40, 13.77)	0.33	4.67 (2.93,17.58)	4.29 (1.38, 12.3)	0.24

Data presented as median (IQR) and analysed using a Mann Whitney U test

Abbreviations: CXCL10 – C-X-C motif chemokine 10/ Interferon gamma-induced protein 10, CCL2 – chemokine (C-C motif) ligand 2, CXCL13 – chemokine (C-X-C motif) ligand 13, CXCL9 – Chemokine (C-X-C motif) ligand 9, CCL5(RANTES) – Chemokine (C-C motif) Ligand 5 (regulated on activation, normal T cell expressed and secreted), IL10 – Interleukin 10, IL4 – Interleukin 4, IL13 – Interleukin 13, APRIL – A proliferation-inducing ligand, BAFF – B lymphocyte stimulator, MMP-9 – Matrix metallopeptidase 9, CRP – C -reactive protein, C5/C5a – Complement component, CSF – Cerebral spinal fluid , ND – Not detected
